# Supplementary material for: Polar Metabolites Profiling of Wheat Shoots (Triticum aestivum L.) under Repeated Short-Term Soil Drought and Rewatering
Source: Int J Mol Sci. 2023 May 8;24(9):8429. doi: 10.3390/ijms24098429 (PMC10179269; doi:10.3390/ijms24098429)
Supplement: Supplementary file 1 [file ijms-24-08429-s001.zip › ijms-2391555-supplementary.pdf]

Supplementary materials

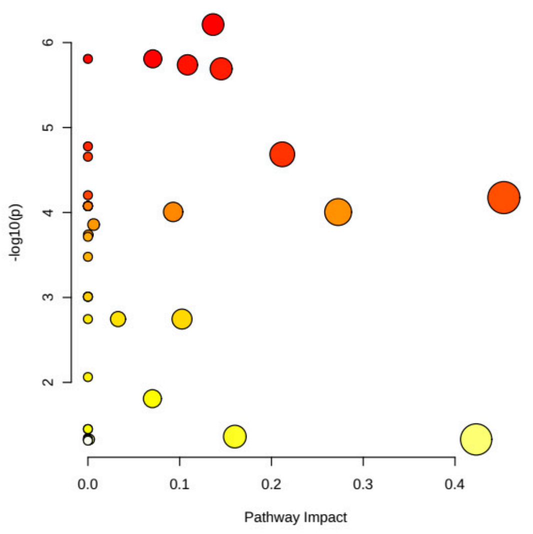

Figure S1. Summary of pathway analysis of differentially accumulated polar metabolites after wheat plants exposition to drought in comparison to control performed using MetaboAnalyst 5.0 platform.

Table S1. Results from pathway analysis of differentially accumulated polar metabolites after wheat plants exposition to drought in comparison to control performed using MetaboAnalyst 5.0 platform.

	Total Cmpd	Hits	Raw p	-log10(p)	Holm adjust	FDR	Impact
Butanoate metabolism	17	1	6.14E-07	6.21E+00	2.33E-05	1.55E-05	0.14
Glutathione metabolism	27	1	1.55E-06	5.81E+00	5.75E-05	1.55E-05	0.07
Thiamine metabolism	22	1	1.55E-06	5.81E+00	5.75E-05	1.55E-05	0.00
Glyoxylate and dicarboxylate metabolism	29	4	1.83E-06	5.74E+00	6.40E-05	1.55E-05	0.11
Citrate cycle (TCA cycle)	20	2	2.04E-06	5.69E+00	6.92E-05	1.55E-05	0.15
Propanoate metabolism	20	1	1.67E-05	4.78E+00	5.50E-04	1.05E-04	0.00
Glycine, serine and threonine metabolism	33	2	2.07E-05	4.68E+00	6.62E-04	1.05E-04	0.21
Amino sugar and nucleotide sugar metabolism	50	2	2.20E-05	4.66E+00	6.83E-04	1.05E-04	0.00
Arginine biosynthesis	18	3	6.26E-05	4.20E+00	1.88E-03	2.13E-04	0.00
Alanine, aspartate and glutamate metabolism	22	6	6.68E-05	4.18E+00	1.94E-03	2.13E-04	0.45
Monobactam biosynthesis	8	1	8.40E-05	4.08E+00	2.35E-03	2.13E-04	0.00
Cysteine and methionine metabolism	46	1	8.40E-05	4.08E+00	2.35E-03	2.13E-04	0.00
Lysine biosynthesis	9	1	8.40E-05	4.08E+00	2.35E-03	2.13E-04	0.00
beta-Alanine metabolism	18	1	8.40E-05	4.08E+00	2.35E-03	2.13E-04	0.00
Nicotinate and nicotinamide metabolism	13	1	8.40E-05	4.08E+00	2.35E-03	2.13E-04	0.00
Starch and sucrose metabolism	22	2	9.85E-05	4.01E+00	2.35E-03	2.21E-04	0.09
Galactose metabolism	27	5	9.88E-05	4.01E+00	2.35E-03	2.21E-04	0.27
Carbon xation in photosynthetic orgnisms	21	3	1.39E-04	3.86E+00	2.92E-03	2.93E-04	0.01
Glycolysis / Gluconeogenesis	26	2	1.83E-04	3.74E+00	3.67E-03	3.67E-04	0.00
Pyruvate metabolism	22	2	1.93E-04	3.71E+00	3.67E-03	3.67E-04	0.00
Cyanoamino acid metabolism	26	3	3.32E-04	3.48E+00	5.98E-03	6.01E-04	0.00
Purine metabolism	63	1	9.81E-04	3.01E+00	1.67E-02	1.55E-03	0.00
Pyrimidine metabolism	38	1	9.81E-04	3.01E+00	1.67E-02	1.55E-03	0.00
Nitrogen metabolism	12	1	9.81E-04	3.01E+00	1.67E-02	1.55E-03	0.00
Inositol phosphate metabolism	28	1	1.80E-03	2.75E+00	2.51E-02	2.53E-03	0.10
Phosphatidylinositol signaling system	26	1	1.80E-03	2.75E+00	2.51E-02	2.53E-03	0.03
Ascorbate and aldarate metabolism	18	1	1.80E-03	2.75E+00	2.51E-02	2.53E-03	0.00
Selenocompound metabolism	13	1	8.65E-03	2.06E+00	9.52E-02	1.17E-02	0.00
Tyrosine metabolism	18	1	1.55E-02	1.81E+00	1.55E-01	2.04E-02	0.07
Valine, leucine and isoleucine biosynthesis	22	3	3.54E-02	1.45E+00	3.19E-01	4.34E-02	0.00
Valine, leucine and isoleucine degradation	37	3	3.54E-02	1.45E+00	3.19E-01	4.34E-02	0.00
Arginine and proline metabolism	28	3	4.34E-02	1.36E+00	3.19E-01	4.82E-02	0.16
Aminoacyl-tRNA biosynthesis	46	10	4.52E-02	1.34E+00	3.19E-01	4.82E-02	0.00
Phenylalanine metabolism	12	1	4.69E-02	1.33E+00	3.19E-01	4.82E-02	0.42
Phenylalanine, tyrosine and tryptophan biosynthesis	22	1	4.69E-02	1.33E+00	3.19E-01	4.82E-02	0.00
Phenylpropanoid biosynthesis	35	1	4.69E-02	1.33E+00	3.19E-01	4.82E-02	0.00
Tropane, piperidine and pyridine alkaloid biosynthesis	8	1	4.69E-02	1.33E+00	3.19E-01	4.82E-02	0.00
Pantothenate and CoA biosynthesis	23	1	4.84E-02	1.31E+00	3.19E-01	4.84E-02	0.00
